# The Role of the Size of the Small Group and Informal Subgroups in Intragroup Conflicts

**DOI:** 10.3390/bs10050084

**Published:** 2020-04-28

**Authors:** Andrey V. Sidorenkov, Eugene F. Borokhovski

**Affiliations:** 1 Academy of Psychology and Educational Sciences, Southern Federal University, 105/42 Bolshaya Sadovaya Str., Rostov-on-Don 344006, Russia; 2 Centre for the Study of Learning and Performance (CSLP), Concordia University, 1515 St. Catherine Street West, S-GA-2.126, Montreal, QC H3G 1W1, Canada

**Keywords:** conflict levels, interpersonal conflict, individual–subgroup conflict, individual–group conflict, subgroup–subgroup conflict, subgroup–group conflict, conflict types, activity-oriented conflict, subject-oriented conflict

## Abstract

This study examines the relationship between (a) the size of groups and the number of informal subgroups in them with conflicts in the context of the group, and (b) the size of the informal subgroups with conflicts in the context of the subgroup. A multidimensional model of intragroup conflict was used, which includes two dimensions: five levels of conflict (i.e., interpersonal (individual–individual), micro-group (individual–subgroup), group (individual–group), subgroup–subgroup, and subgroup–group) and two types of conflict (activity-oriented and subject-oriented). Each level of conflict contains two types. Forty-one small work groups (334 employees) took part in the study. In the group as a whole, both the size of the group and the number of informal subgroups in it have a positive relationship with subgroup–subgroup conflict in both types and subgroup–group subject-oriented conflict, and have a negative connection with micro-group subject-oriented conflict. In turn, the size of the subgroup is positively associated with the two types of subgroup–group conflict and negatively connected with the two types of micro-group conflict, as well as with interpersonal activity-oriented conflict at the level of the informal subgroup.

## 1. Introduction

Conflict in small working groups, as is well-known, is a complex phenomenon; therefore, the analysis of all of the sides of conflict will allow a better understanding of its role in the activeness of groups and their members including the processes and results of their joint and individual work, the mental state of the employees, their work and group satisfaction, etc. The conceptualization of the multidimensional nature of conflict, in general, is confined to the idea of the types of interpersonal conflict, such as cognitive conflict, affective conflict [1], relationship conflict, task conflict, and process conflict [2,3,4]. Occasionally, they mention the multilayer nature of interpersonal conflict, considering its individual and group levels [5]. The first one happens between certain members, is perceived by the person during direct interaction with other members, and is defined by interpersonal peculiarities. The second one is related to the acknowledgement of all of the conflict members in the group in general, is the consequence of its aggregated perception by the members, and is defined by the group characteristics. Overall, the study of intragroup conflict is limited by interpersonal conflict [6]. Nevertheless, there may be not only interpersonal conflicts in the group, but individual–group conflicts, individual–subgroup conflicts, etc. In particular, in working groups (departments, teams, working shift groups, small companies, etc.) there exist informal groups [7], and therefore there exist conflicts between a person and a subgroup, between subgroups, and between a group and a subgroup. An informal subgroup is a collection of members of a small group that are united on the basis of one or more general psychological characteristics that are significant for them compared to other members of the group. Informal subgroups, as a rule, arise (are formed) spontaneously. It was found that individual–subgroup conflict was much weaker than either interpersonal conflict or individual–group conflict within a group [7].

The topical issue is to find out what has caused a conflict and under what circumstances it unfolds (escalates or subsides). It is a necessary step in conflict modeling and management. Specifically, this can include: the contradictions [8]; personal characteristics of group members [9,10]; leadership [11]; psychological characteristics of the group (for instance, trust [12] and cohesion [13]); group quantitative characteristics, like composition [14,15] and size [16]; and others. Among other factors, one of the studies describes a positive connection between the size of the group and cognitive and affective conflict [16], whereas another one does not reveal a clear connection between the number of persons in a group and the relationship and task conflict in it [17]. We suggest that it would be promising to study the connection of the group size and other group features not only with interpersonal conflict but with other aspects (levels) of intragroup conflicts as well, including the conflicts between a person and a group, a person and an informal subgroup within a group, etc.

**Figure 1:**
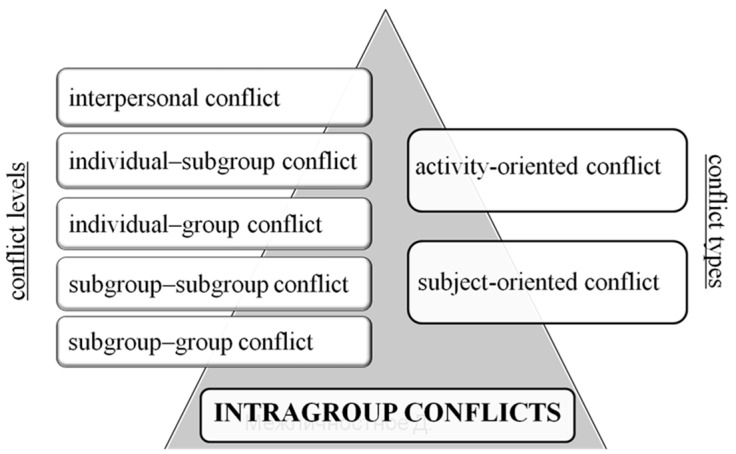
Types and levels of intragroup conflict.

The current study applies a multidimensional model of intragroup conflict [18,19,20]. Conflict is defined as being created by the intensification of the antagonism form of disintegrative interaction and relation between subjects (a person, a subgroup, or a group), revealed in their active negativity to each other [18]). It comprises two dimensions: levels and types (Figure 1). The conflict levels include: (1) *interpersonal* (i.e., between group members inside the informal group, between the members of different subgroups, etc.); (2) *micro-group* or *individual–subgroups* (i.e., between an individual and the informal subgroup in a group); (3) *group* or *individual–group* (i.e., between a group member and the entire group); (4) *inter-subgroup* or *subgroup–subgroup* (i.e., between subgroups within the same group); and (5) *subgroup–group* (i.e., between a subgroup and the entire group).

In this case, we understand the levels as the conflicts expressed in the connections between different subjects (interpersonal, individual–group, subgroup–subgroup, etc.). There may be a conflict between group members, for example, about the ways to solve a problem or revolving around the issue of personal hostility (individual–individual conflict). It can occur between with individual group member, on the one hand, and a subgroup/group, on the other (individual–subgroup/individual–group conflict, respectively), for example, if a person opposes the opinion of a subgroup/group or does not comply with the group norms/demands. A conflict can arise between subgroups within the group (subgroup–subgroup conflict) when subgroups defend their members, defend their interests, or compete among themselves for dominance (high status) in the group, etc. It can also unfold between a subgroup and a group (subgroup–group conflict), when a subgroup imposes its position on the group or the group seeks to weaken the influence of the subgroup. Such a vision of the conflict levels differs from the existing point of view: the individual perception of conflict with another person (the individual level), or conflict between the members of the group in general (the group level) [5]. Each of the five levels of the conflict can be presented as two types: activity-oriented conflict (AOC) and subject-oriented conflict (SOC) [7]. The major criterion distinguishing the two is the corresponding area of group activity–either instrumental (directly linked to any task-related joint activities) or social (interpersonal contacts and relationships outside of task-related activities). Thus, AOC can be described as a conflict in which confrontation between/among its sides is rooted in task-related group activities. On the other hand, SOC encompasses negative perceptions and confrontational actions that are not directly connected to task-related behaviors. SOC does not stem from the professional or business-aligned characteristics of the sides involved in a conflict but is rooted in their subjective qualities such as personal traits; the norms and values of the individuals, subgroups, and the entire group; the conflict participants’ behavioral patterns; and so on.

Thus, it is important to study the relationships of the group size not only with interpersonal conflict, but also with other levels of intragroup conflict, as indicated earlier (according to its two types: activity-oriented conflict and subject-oriented conflict). In addition, it is imperative to understand the connection between the number of informal subgroups in a group and certain levels of conflict. It is reasonable to assume that the larger the group is, the more the subgroups that could be formed within it. Such an increase in the group size and, subsequently, in the number of subgroups will lead to intensifying, first of all, conflicts between subgroups as well as between a subgroup and the group as a whole. Individual group members included in various subgroups often build relationships between themselves and the entire group based on their association with the respective subgroup and in the interests of this subgroup. Therefore, when contradictions in a group become aggravated, they often tend to lead to conflicts between various subgroups or between a subgroup and the entire group rather than to interpersonal conflicts. Moreover, interpersonal conflict (a conflict between members of different subgroups) often quickly grows into a conflict between the respective subgroups or between a subgroup and the entire group, when the majority of the group members take the side of one particular subgroup. One could also assume a negative relationship between the size of a group and the number of subgroups within it, with micro-group conflict, as individual group member(s) not included in any subgroup may be afraid to conflict with a “foreign” subgroup, since they realize its advantage and strength as a collective subject.

In the context of an informal subgroup, there should be negative relationships between the size of the subgroup, on the one hand, and interpersonal and micro-group conflicts, on the other. As the size of a subgroup increases, the likelihood of these conflicts arising inside it also increases. In order to ensure the integrity of the subgroup and stability of its functioning, the members of the subgroup should strive not to conflict with each other and with their own subgroup. Only in this case can they guarantee that the subgroup will successfully interact with other subgroups and with the group as a whole.

This study examines the relationship between (a) the size of group and the number of informal subgroups in it with conflicts in the context of the group, and (b) the size of the informal subgroup with conflicts in the context of the subgroup.

The hypotheses of the research are:

**Hypothesis** **1.** *Group size and the number of informal subgroups within a group are positively associated with conflicts among subgroups and between a subgroup and the group as a whole.*

**Hypothesis** **2.** *Group size and the number of informal subgroups within a group are negatively associated with individual–subgroup conflicts.*

**Hypothesis** **3.** *The size of the informal subgroup is negatively related to interpersonal conflict and micro-group conflict within a subgroup.*

## 2. Methods

### 2.1. Participants

We studied 41 groups—small companies and primary business units in middle-sized and larger Russian organizations. The groups differ in their field of activity: trade, social services, banking, projects and design, production, etc. The number of employees in groups varied between 4 and 21 (*M* = 8.0). The total number of participants was 334. The sample was composed of 62.2% of women and 37.8% of men, aged between 19 and 70 years old (*M* = 30). The employment time of the people in the groups under investigation was between 1 and 300 months (*M* = 71.5).

### 2.2. Instruments

The informal subgroups in small groups were determined with a special formalized algorithm [21]. The following actions compose this algorithm: (a) the “description” matrix that characterizes the concrete status of the combined variables is produced; (b) the numerical values of the links (degrees of similarity) among the grouped members are determined, and subsequently, the similarity factor matrix is constructed; (c) individuals are organized into subgroups according to the index, which characterizes the quality of these subgroups (the grouping procedure identifies which member of a small group is regarded as the “center” of a possible subgroup, and the subgroup composition is determined in a way that ensures the highest possible “density” of such a subgroup); and (d) subgroups with the highest “density” are selected [22].

The study employed Russian versions of two questionnaires for assessing intragroup conflicts, as follows. The interpersonal conflicts questionnaire has 8 items for measuring the relevant activity-oriented conflicts (AOC-Inter-P) and subject-oriented conflicts (SOC-Inter-P) (e.g., “Discussion of how to complete the tasks set by the group leader is often accompanied by open confrontation among colleagues” and “Colleagues are often unfriendly to each other”) [19]. The questionnaire consists of two parts (“In the group, as a whole” and “Those I keep close relations with”) to assess interpersonal conflict in a group (the first part) and within informal subgroups (the second part). Evaluation by the subjects of the severity of the attribute reflected by each item is on the basis of a seven-point scale (from completely agree = 1 to completely disagree = 7). The Cronbach’s alpha values were 0.89 (AOC) and 0.90 (SOC). The questionnaire for the group and micro-group conflict also includes 8 items to measure the individual–group (AOC-IG and SOC-IG) and individual–subgroup (AOC-IS and SOC-IS) levels of conflicts [20]. It has two parts, “The group as a whole” and “The unity of those with whom I keep close relation”, to measure the two types, respectively—the group conflict (the first part) and the micro-group conflict (the second part). Some examples of items are: “The group […] often sharply criticizes one of its members for how they perform professional duties” and “The group […] often openly and harshly expresses opinions about one of its members”. Each item is responded to twice—firstly, with respect to the group as a whole and then with respect to a unity of those with whom the respondent maintains close relationships—according to the questionnaire structure.

Response options were arranged alongside a 7-point scale. The Cronbach’s alpha values for the subscales were 0.91 (AOC-IG) and 0.96 (SOC-IG).

The evaluation of the conflict between the subgroups (AOC-SS and SOC-SS) and between a subgroup and a group (AOC-SG and SOC-SG) was done with the relative indicators:

$SS=\frac{\mathrm{Inter}\;-\;P\;group}{\mathrm{Inter}\;-\;P\;subgroup}10$, where *SS* = subgroup–subgroup conflict, Inter-P *group* = the evaluation of the interpersonal conflict (according to the relevant type) in the part “In the group as a whole”, Inter-P *subgroup* = that in the part “With whom I keep close relation”, and 10 = constant.

$SG=\frac{IG}{IS}10$, where *SG* = subgroup–group conflict, *IG* = the evaluation of the individual–group conflict, *IS* = individual–subgroup conflict (according to the relevant type), and 10 = constant.

In the foundation of this method for the assessment of subgroup–subgroup conflict lies the following consideration being its the basis: the greater the difference between the interpersonal conflict within the group (primarily between members of different subgroups) and the interpersonal conflict within the subgroups—that is, the stronger the former compared to the latter—the higher the likelihood that there will be a stronger subgroup–subgroup conflict. Nearly the same logic was used in evaluating subgroup–group conflict: the stronger the individual–group conflict compared to the individual–subgroup conflict, the more pronounced the conflict between the respective subgroup and the entire group. In other words, the higher the conflict of individual members of the subgroup with the group as a whole and the less with their own subgroup, the more likely the conflict between this subgroup and the entire group is to intensify.

### 2.3. Procedure

The questionnaires used were interrelated components of a computerized psycho-diagnostic system “Group Profile” (GP) [22], a modification of 2016 (GP-M16) with a function of maintaining control over the standardized data collection protocol and an increased capacity for data analyses. The system executed first-level data analysis, checking for the consistency and reliability of the data collection process. Specifically, GP-M16 could suspend accepting respondents’ input if frequent omissions and/or apparently biased responses were detected. Study participants worked on personal computers equipped with GP-M16 individually.

### 2.4. Statistical Methods

Linear regression analysis was employed to assess the degree of association of the group size with levels and types of intragroup conflict (as the criterion variables). Regression analysis was performed within the SPSS 17.0 software package.

## 3. Results

### 3.1. Informal Subgroups in a Group

There were 70 subgroups in total; their numbers in groups varied between 1 and 4. Two hundred and fifteen employees (66.6%) were included in subgroups. Between 14.5% and 100% of employees in the different groups constituted the subgroups. The most numerous were dyads (38.6% of subgroups) and triads (31.4%), whereas other subgroup sizes were much less prevalent: 17.2% of subgroups were composed of four members, 10.0% of five members, and 2.8% of six members.

The fact that informal subgroups arise within a group determines the possibility of the following levels of conflict: individual–subgroup, subgroup–subgroup, and subgroup–group. Our results suggest that within the groups, conflicts with high probability may occur between a person and a subgroup, between subgroups, and between a subgroup and the entire group. In addition, it has been found that the number of informal subgroups depends on the size of the group (r = 0.73, *p =* 0.000), that is, the larger the group is, the more subgroups it has.

To increase the precision of the analysis in the subgroup context, we have excluded the results of 15 subgroups, each of those having been the only one in its group. That decision was grounded by the fact that in such subgroups, a priori, there will be no conflicts between subgroups, and the conflict between a subgroup and a group is unlikely to happen if the majority of the group is included into the only subgroup. For example, if the majority of members (for example, five people out of seven group members) are included in an informal subgroup, then how can a conflict arise between this subgroup and the group as a whole? Would that mean a conflict of the subgroup with itself? It cannot be. Individual members of a subgroup may conflict with each other, but it will be a case of interpersonal conflict, and not a conflict between a subgroup and a group. Some of the subgroup members may conflict with a member not included in this subgroup, but this conflict would also be classified as interpersonal. Thus, 55 subgroup results were retained for the analysis.

### 3.2. The Connection of the Group Size and the Number of Subgroups with the Components of the Intragroup Conflict

In the whole group, both the group size and the number of informal subgroups are positively connected with conflict between subgroups in two conflict types, and subgroup–group subject-oriented conflict (Table 1). The group size is also positively associated with subgroup–group activity-oriented conflict, and the number of subgroups is related to individual–group activity-oriented conflict. Besides, the group size and the number of subgroups in it are negatively related to individual–subgroup subject-oriented conflict.

Therefore, the first and second hypotheses are largely confirmed. However, no significant relationship was found between group size and the number of subgroups with individual–subgroup activity-oriented conflict (as would have been expected according to Hypothesis 2). We also found a positive relationship between the number of subgroups and individual–group activity-oriented conflict, which we did not anticipate.

### 3.3. Connection between the Subgroup Size and the Conflict Components in the Context of the Informal Subgroup

The size of the subgroup is positively associated with the two types of subgroup–group conflict and negatively connected with the two types of individual–subgroup conflict, as well as with interpersonal activity-oriented conflict at the level of the informal subgroup (Table 2).

Thus, the third hypothesis has been confirmed with the exception of interpersonal subject-oriented conflict. Besides, we have additionally revealed a connection between the subgroup size and the subgroup-group conflict.

## 4. Discussion

No association of group size with any type of interpersonal conflict was found, which is consistent with the findings of K.A. Jehn [17]. At the same time, the enlargement of the group and the number of informal subgroups in it causes the intensification of subgroup–subgroup conflict and subgroup–group conflict and, on the contrary, leads to the weakening of individual–subgroup conflict at the group level. Those connections could be explained as follows. With an increase in group size, the number of subgroups included in them also grows. Their participation in the life of the group also becomes more intense. The subgroups become more important subjects of activeness and relationships within the group. They strongly influence the group life, as they fulfill certain functions related to their members (to support in achieving personal goals and in meeting social needs, to provide a feeling of security, to create the rules inside the group and monitor their observation, etc.) and in the group as a whole (to regulate the intragroup’s activeness and to solve the group’s problems and others) [18]. With an increase in the number of informal subgroups, their role in the group life grows as well; therefore, the natural consequence is the intensification of subgroup–subgroup and subgroup–group conflicts. On the other hand, an increase in the group size and the number of subgroups in the group leads to an increasing importance of the relationships with subgroups for some members, and that is why the micro-groups should be carefully considered. Some members are more careful and cautious not to get involved in a conflict with any subgroup. It naturally defuses the individual–subgroup conflict.

A somewhat similar situation can be observed in the context of the subgroup. That is, an increase in the number of members of the subgroup intensifies subgroup–group conflict and relieves individual–subgroup conflict. The difference is in the decrease in interpersonal conflict in a subgroup related to its size increase. We could suggest that the fewer the people that get in conflict with each other and their subgroup, the more united and stabilized the subgroup is. That means it has more opportunities to interact successfully with other subgroups and the group itself. That is why the decrease in interpersonal conflict and individual–subgroup conflict, depending on the subgroup size, can be regarded as a sort of protective response. The probability of conflict between the subgroup and the non-member (individual–subgroup conflict) also decreases as the size of that subgroup increases. This tendency stems from the fact that people who are not members of the subgroup will be cautious or even afraid to come into conflict with a large subgroup, since it is obvious that “the forces are not even”. Besides, the more numerous the subgroup is, the more possibilities it has for a successful conflict resolution with a group in general. Its members are more confident in the positive resolution of the conflict and, respectively, are more prepared for the group conflict than the members of a smaller subgroup (a dyad, for instance) within a large-size group. That is why we can state the existence of the positive connection between the subgroup size and subgroup–group conflict.

## 5. Conclusions

The model of intragroup conflicts, that we proposed here, goes beyond the tradition of studying only interpersonal conflict. Scientists can study the precursors (including group size, composition, etc.) and the consequences of conflicts of different levels (not limited to interpersonal conflict) and types. Research can focus on the interconnections and transformations of not only conflict types (e.g., relationship conflict and task conflict) [23,24], but also conflict levels (e.g., interpersonal, individual–subgroup, etc.). For example, we can assume that the emergence and further strengthening or weakening of a certain level of conflict (e.g., interpersonal) may lead to the appearance, and subsequent increase or decrease in, conflicts on other levels (e.g., subgroup–subgroup).

Practitioners (administrators, business-consultants, etc.) can predict levels and types of conflict based on knowledge of their precursors. They can foresee the consequences of one or other level of conflict, given that the psychological science has accumulated the corresponding knowledge. For example, based on the multidimensional model of intragroup conflict and respective research findings, specialists in the field can more effectively not only predict but also manage intragroup conflicts by manipulating group size. Specifically, reducing it should lead to a decrease in subgroup–subgroup and subgroup–group conflicts of both activity-oriented and subject-oriented types. It is generally accepted that interpersonal relationship conflict (similarly to subject-oriented conflict) is usually dysfunctional [3,25], may lead (increase motivation) to quitting the current job [26], and therefore should be reduced. A moderate task conflict (similar to activity-oriented conflict) often positively affects a team’s work performance [3,17], especially if the team members are engaged in complex cognitive tasks. However, the same conflict may have negative consequences [3,17,27], especially in groups that perform simple executive tasks. The results also showed that process conflict negatively affects group performance, member satisfaction, and group coordination [28].The ambiguity of the data about relationship conflicts and task conflicts stimulated interest in the contingency model. Specifically, a number of variables that moderate the relationships between conflict and team performance have been identified, for example, team goal orientation, learning orientation, and performance orientation [29], emotion regulation [30].

Our study did not reveal a connection between group size and interpersonal conflict. Nevertheless, the identified consequences of the two types of conflict at the interpersonal level could hypothetically be projected onto subgroup–subgroup and subgroup–group conflicts. A decision by the experts of whether to weaken or intensify the conflict depends on a number of circumstances (content of the joint activity, type of the task, degree of interdependence of the group members, etc.). Normally, in most groups, strong subgroup–subgroup and subgroup–group conflict of both types should be reduced. In conclusion, we would like to note that this study is just the first step towards expanding our understanding of the causes of, consequences of, and relationships between different levels (not only interpersonal) of intragroup conflict.

## Figures and Tables

**Table 1:** Results of regression analysis of associations between the size and number of informal subgroups (independent variables) and conflict levels and types (dependent variables) in the group as a whole.

Group Characteristics	Model Statistics	Conflict									
		AOC-IP	SOC-IP	AOC-IS	SOC-IS	AOC-IG	SOC-IG	AOC-SS	SOC-SS	AOC-SG	SOC-SG
Size	*β*	0.12	0.04	−0.28	−0.42 **	0.23	0.09	0.51 *	0.54 *	0.46 *	0.64 **
	*R* ^2^	0.01	0.00	0.08	0.18	0.05	0.00	0.14	0.13	0.10	0.20
Number of subgroups	*β*	0.31	−0.22	−0.39	−1.57 *	1.18 *	0.21	2.38 *	2.31 *	1.90	2.28 *
	*R* ^2^	0.01	0.00	0.01	0.11	0.14	0.00	0.13	0.11	0.08	0.11

Note: **—*p* < 0.01, *—*p* < 0.05.

**Table 2:** Results of regression analysis of associations between informal subgroup size (independent variables) and conflict levels and types (dependent variables) in the context of the subgroup.

Characteristic of Subgroups	Model Statistics	Conflict									
		AOC-IP	SOC-IP	AOC-IS	SOC-IS	AOC-IG	SOC-IG	AOC-SS	SOC-SS	AOC-SG	SOC-SG
Size	*β*	−0.79 *	−0.54	−1.5 **	−0.97 *	−0.16	0.25	0.76	0.57	1.55 *	1.69 *
	*R^2^*	0.07	0.04	0.16	0.08	0.00	0.00	0.02	0.01	0.09	0.11

Note: **—*p* < 0.01, *—*p* < 0.05.

## Abbreviations

AOC-Inter–P	interpersonal activity-oriented conflict,
SOC-Inter–P	interpersonal subject-oriented conflict,
AOC-IS	individual–subgroup activity-oriented conflict,
SOC-IS	individual–subgroup subject-oriented conflict,
AOC-IG	individual–group activity-oriented conflict,
SOC-IG	individual–group subject-oriented conflict,
AOC-SS	subgroup–subgroup activity-oriented conflict,
SOC-SS	subgroup–subgroup subject-oriented conflict,
AOC-SG	subgroup–group activity-oriented conflict,
SOC-SG	subgroup–group subject-oriented conflict.
